# Momordicoside G Regulates Macrophage Phenotypes to Stimulate Efficient Repair of Lung Injury and Prevent Urethane-Induced Lung Carcinoma Lesions

**DOI:** 10.3389/fphar.2019.00321

**Published:** 2019-03-29

**Authors:** Zhenhua Du, Shuhui Zhang, Yukun Lin, Lin Zhou, Yuehua Wang, Guixi Yan, Mengdi Zhang, Mengqi Wang, Jiahuan Li, Qiaozhen Tong, Yongjian Duan, Gangjun Du

**Affiliations:** ^1^Institute of Pharmacy, College of Pharmacy, Henan University, Kaifeng, China; ^2^School of Pharmacy and Chemical Engineering, Zhengzhou University of Industrial Technology, Xinzheng, China; ^3^College of Pharmacy, Hunan University of Chinese Medicine, Changsha, China; ^4^Department of Oncology, The First Affiliated Hospital of Henan University, Kaifeng, China

**Keywords:** momordicoside G, macrophage phenotypes, lung injury, carcinoma lesions, injury repair

## Abstract

Momordicoside G is a bioactive component from *Momordica charantia*, this study explores the contributions of macrophages to the effects of momordicoside G on lung injury and carcinoma lesion. *In vitro*, when administered at the dose that has no effect on cell viability in M2-like macrophages, momordicoside G decreased ROS and promoted autophagy and thus induced apoptosis in M1-like macrophages with the morphological changes. In the urethane-induced lung carcinogenic model, prior to lung carcinoma lesions, urethane induced obvious lung injury accompanied by the increased macrophage infiltration. The lung carcinoma lesions were positively correlated with lung tissue injury and macrophage infiltration in alveolar cavities in the control group, these macrophages showed mainly a M1-like (iNOS^+^/CD68^+^) phenotype. ELISA showed that the levels of IL-6 and IL-12 were increased and the levels of IL-10 and TGF-β1 were reduced in the control group. After momordicoside G treatment, lung tissue injury and carcinoma lesions were ameliorated with the decreased M1-like macrophages and the increased M2-like (arginase^+^/CD68^+^) macrophages, whereas macrophage depletion by liposome-encapsulated clodronate (LEC) decreased significantly lung tissue injury and carcinoma lesions and also attenuated the protective efficacy of momordicoside G. The M2 macrophage dependent efficacy of momordicoside G was confirmed in a LPS-induced lung injury model in which epithelial closure was promoted by the transfer of M2-like macrophages and delayed by the transfer of M1-like macrophages. To acquire further insight into the underlying molecular mechanisms by which momordicoside G regulates M1 macrophages, we conduct a comprehensive bioinformatics analysis of momordicoside G relevant targets and pathways involved in M1 macrophage phenotype. This study suggests a function of momordicoside G, whereby it selectively suppresses M1 macrophages to stimulate M2-associated lung injury repair and prevent inflammation-associated lung carcinoma lesions.

## Introduction

In spite of revised and aggressive approaches to therapy, the five-year survival rate for advanced stage lung cancer has not improved significantly (Markovic et al., 2008). Carcinogenesis is associated with intrinsic cellular changes and inflammatory factors in the tumor microenvironment (Witz, 2008). Epidemiological and preclinical studies have shown that whole food-derived components are associated with reduced risks of cancers (Stan et al., 2008). Recently, *Momordica charantia* has gained significant attention for its anticancer efficacy and its active components against various cancers (Raina et al., 2016). As an edible fruit and folk drug, *M. charantia* is extremely good for health and has been used traditionally for various therapeutic benefits in China, Southeast Asia and South America (Grover and Yadav, 2004). Research over decades has progressively investigated pharmacological actions of *M. charantia*, and the bioactive compounds isolated from *M. charantia* have shown significant anti-cancer activity in cancer cell lines and xenografted mice by regulation of cancer cell protein network (Farooqi et al., 2018). Chemical analysis shows that momordicoside G is a cucurbitane-type triterpene glycoside in *M. charantia* and such compounds were reported to be able to inhibit breast cancer (Ray et al., 2010), colon cancer (Dia and Krishnan, 2016), brain cancer (Duan et al., 2015) and lung cancer (Wang et al., 2012) *in vitro*. In addition, the extracts of *M. charantia* have shown to reduce DMBA-induced skin papilloma and prevent spontaneous mammary tumorigenesis in mice (Ganguly et al., 2000; Nagasawa et al., 2008). Sur et al. (2018) reported that bitter melon prevented the development of 4-NQO-induced oral squamous cell carcinoma by modulating immune signaling. Yang et al. (2018) found that *M. charantia* extract exerted its anti-inflammatory activity in murine macrophages by reducing the action of TAK1 and affecting the activation of NF-κB and AP-1. “Cancer is a never cured wound” (Rosowski and Huttenlocher, 2015). In our previous study, wound healing could prevent carcinogenesis (Liu et al., 2015). It has been proved that *M. charantia* can be effectively used to treat diabetic wounds (Hussan et al., 2014; Singh et al., 2017). However, whether these pharmacological actions of *M. charantia* are associated with its active component momordicoside G remains unknown, and a comprehensive study on momordicoside G and its beneficial attributes is also lacking. It is well known that macrophages play a key role in tissue injury repair (Gensel and Zhang, 2015; Wynn and Vannella, 2016). Here, we investigated whether momordicoside G can promote wound healing against carcinogenesis and explored the contributions of macrophages to the effects of momordicoside G on lung injury and carcinoma lesion.

## Materials and Methods

### Materials

Momordicoside G (purity > 98% via HPLC) from *M. charantia* was purchased from Lianshuo Biotechnology Co., Ltd. (Shanghai, China). RPMI 1640 was obtained from Cellgro (Mediatech, United States). Fetal bovine serum (FBS) was obtained from Hangzhou Sijiqing Biological Engineering Materials Co. (China). Urethane, LPS, DAPI, CFSE, IL-10, AO, and Evans blue were purchased from Sigma Chemical Co. (St. Louis, MO, United States). MTT, DMSO were obtained from Life Technologies (United States). Antibodies used herein including anti-NLRP3, anti-arginase, anti-CD68, anti-iNOS, anti- LC3-B, anti-p62, β-actin, and FITC-conjugated goat anti-mouse IgG were obtained from BD Pharmingen. HRP-conjugated goat anti-mouse IgG polyclonal antibody, Annexin V-FITC Apoptosis kit, ROS, DCFH-DA and mouse quantitative ELISA kits (IL-6, IL-12, IL-10, and TGF-β1) were obtained from R&D Systems. Nitric oxide (NO) assay kit was obtained from Nanjing Jiancheng Bioengineering Institute. Standard rodent chow was purchased from Henan Provincial Medical Laboratory Animal Center (Zhengzhou, China), License No. SCXK (YU) 2015-0005, Certificate No. 41000100002406.

### Animals

Six-week-old female ICR mice were obtained from Henan Provincial Medical Laboratory Animal Center. All mice were housed in individual ventilated cages (lights on 7:00 AM to 7:00 PM). Animals were fed standard rodent chow and water. All animal procedures were approved by the Animal Experimentation Ethics Committee of Henan University (permission number HUSAM 2016-288), and all procedures were performed in strict accordance with the Guide for the Care and Use of Laboratory Animals and the Regulation of Animal Protection Committee to minimize suffering and injury. Animals were euthanized via carbon dioxide overdose based on experimental need.

### Cell Culture and Assay

The Raw264.7 macrophages were from ATCC, purchased from the Chinese Academy of Sciences and grown in RPMI 1640 medium supplemented with 10% (v/v) fetal bovine serum (FBS) in a humidified atmosphere containing 5% CO_2_ and 95% air at 37°C. Macrophages were seeded in 24-well plates and stimulated by 10 ng/ml LPS or 10 ng/ml IL-10 for 24 h to obtain M1-like (iNOS^+^) and M2-like (arginase^+^) macrophages, subsequently washed once with PBS and cultured in new medium containing different concentrations (10–40 μM) of momordicoside G for another 24 h. The supernatant was then collected for assays of IL-12, IL-10, TGF-β1, ROS, and NO. IL-12, IL-10, and TGF-β1 were tested by ELISA kits, ROS was determined by DCFH-DA, and NO was determined by colorimetric assay kit. The results were calculated from linear curves obtained using the quantikine kit standards. Cell proliferation was examined by MTT reduction assay, according to our previous method (Cao et al., 2016).

Cells were analyzed by Laser holographic cell imaging and analysis system (HoloMonitor M4, Phiab, Sweden). The immunophenotypes were analyzed by FITC-conjugated anti-mouse-iNOS or anti-mouse-arginase staining. Cells were stained with AO (1 μg/ml) at 37°C for 30 min before observation. Red acidic vesicular organelles (AVOs) stained by AO in autophagic cells were visualized under a fluorescence microscope (emission wavelength 488 nm/515 nm). Cell autophagy was further estimated by immunofluorescence staining of LC3-B and p62 production using FITC-conjugated anti-LC3-B or anti-p62. The binding of ANXV-FITC to phosphatidylserine was used as a measure of macrophage apoptosis by an automated cell counter and analysis system (Nexcelom Cellometer X2, Nexcelom, United States).

### Urethane-Induced Lung Carcinogenic Model

Urethane (600 mg/kg body weight) alone or in combined with liposome-encapsulated clodronate (LEC, 4 mg/mouse) was injected intraperitoneally (i. p.) into ICR mice once a week for four or eight weeks (twenty mice per group), according to our previous lung carcinogenic model (Ma et al., 2016). Following the first urethane injection, mice received momordicoside G (50 mg/kg/day) once a day via intragastric administration for four or eight weeks. At five and nine weeks after the first urethane injection, ten mice were sacrificed under anesthesia with pentobarbital sodium (90 mg/kg), the alveolar fluid was collected by inserting a cannula into the trachea with three sequential solutions (1 mL PBS), supernatant was used for cytokine assay (IL-6, IL-12, IL-10, and TGF-β1) after centrifugation, and the centrifuged cells were resuspended in 0.9% sterile saline for total cell counts and added into a magnetic cell sorting column for macrophage collection based on anti-CD68-coated beads.

A part of each lung was preserved in 10% buffered formalin and routinely embedded in paraffin. Lung sections were stained by H&E and immunohistochemistry according to our previously described method (Cao et al., 2016). Inflammatory score (IS) is calculated by the area of involved inflammatory infiltration: grade 0, no inflammatory infiltration; grades 1, inflammatory infiltration less than 10% of the scanned fields; grade 2, inflammatory infiltration from 10 to 30% of the scanned fields; grade 3, inflammatory infiltration from 30 to 50% of the scanned fields; grade 4, inflammatory infiltration more than 50% of the scanned fields. Atypical adenomatous hyperplasia is regarded as the lung carcinoma lesions based on the histological appearance.

For NLRP3, the total immunohistochemical score was calculated by the intensity score and proportion score by excluding the primary antibody and IgG matched serum, respectively as positive and negative controls. For macrophage phenotypes, after overnight incubation with the primary antibodies (anti-mouse-iNOS, anti-mouse-arginase and anti-mouse-CD68), slides were incubated with the FITC-conjugated goat anti-mouse IgG for 30 min. An automated scanning system equipped with an Olympus fluorescence microscope was used to determine the positive staining, the total fluorescence intensity was calculated in five successive fields.

Cytokines including IL-6, IL-12, IL-10, and TGF-β1 were determined.

### LPS-Induced Lung Injury Model

Lung injury was induced by 2 mg/kg of LPS via intratracheal injection according to previous method (Su et al., 2014) in macrophage-competent and macrophage-deleted mice (twenty mice per group). The macrophage-competent mice only received momordicoside G after LPS injection. For macrophage-deplation, 3 days before lung injury induction, LEC (4 mg/mouse) was injected intraperitoneally into mice. Following LPS injection, *in vitro* IL-10 or LPS-polarized Raw264.7 macrophages (2 × 10^6^ cells in 200 μL saline) were labeled with CFSE and injected intravenously into mice once a week for two weeks; simultaneously, mice were received momordicoside G (50 mg/kg once a day via intragastric administration) for 2 weeks. Lung function, lung permeability and lung injury were assessed at 2, 8, and 15 days after the lung injury. Lung function was analyzed by maximal mid-expiratory flow using the animal respiratory metabolic measurement system (Sable Systems International, United States). Lung permeability was assessed with the Evans blue dye extra-barrier technique and the wet/dry ratio of lungs according to our previous method (Meng et al., 2017). Lung injury was evaluated by pathohistological examination. At 15 days, alveolar macrophages were isolated from macrophage-competent mice by magnetic cell sorting based on anti-CD68-coated beads, and lung sections from macrophage-deleted mice were observed under a fluorescence microscope. Protein was extracted from isolated alveolar macrophages in cell lysis buffer. Equal amounts of protein were separated via 12% sodium dodecyl sulfate-polyacrylamide gel electrophoresis (SDS-PAGE), electroblotted on nitrocellulose membranes, and probed with antibodies against iNOS, arginase, LC3-B, and P62. Antibody binding was detected via enhanced chemiluminescence, according to the manufacturer’s instructions (Pierce, Rockford, IL, United States). Band density was quantified using ImageJ software (NIH, Bethesda, MD, United States) and normalized to the corresponding control group.

### The Regulatory Mechanism of Momordicoside G on Macrophages

The gene expression profiles GSE5099 was obtained from the Gene Expression Omnibus (GEO) database, up- and downregulated genes related to M1-associated macrophages were identified using GEO2R, and the human structures of these differential proteins were collected from the protein data bank (PDB) for docking. The chemical structure of momordicoside G was obtained from PubChem and the docking exercise was conducted using the online software systemsDock with the auto-removement of non-specified protein structures. Docking scores over 6 was regarded as the potential targets for momordicoside G. The gene ontology (GO) and Kyoto Encyclopedia of Genes and Genomes (KEGG) enrichment analyses were performed for the potential targets using the Database for Annotation, Visualization and Integrated Discovery (DAVID), and the online software Omicshare. The protein-protein interaction (PPI) among these potential targets was constructed using the STRING database and the hub genes were identified using Cytoscape.

### Statistical Analyses

The data was presented as the mean ± SD and statistically analyzed using GraphPad Prism, Version 5.0 (San Diego, CA, United States). The difference between two groups was evaluated using a *t*-test. A *P* value of less than 0.05 was considered significant.

## Results

### Momordicoside G Regulates Macrophages *in vitro*

**FIGURE 1:**
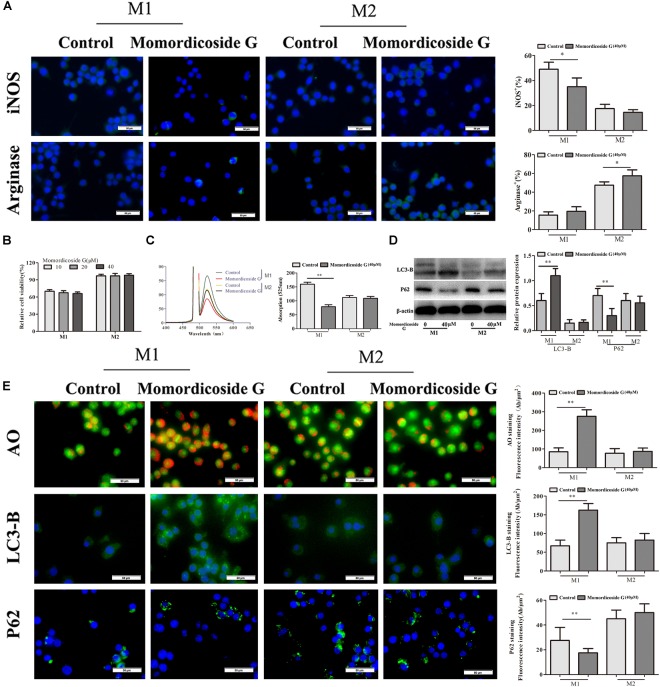
Momordicoside G selectively affects M1 macrophage phenotype and function *in vitro*. **(A)** Macrophage phenotypes analyzed by FITC-conjugated anti-mouse iNOS or arginase staining (*n* = 10, 40×). **(B)** Cell viability examined by MTT (*n* = 5). **(C)** ROS detected by DCFH-DA (*n* = 5). **(D)** Cell autophagy-associated markers examined by Western blot (*n* = 3). **(E)** Cell autophagy indicated by AO, LC3-B, and P62 staining (*n* = 5, 40×). The data presents mean ± SD, the experiments were repeated 3 times, and statistical significance was determined by a *t*-test. ^∗^*P* < 0.05, ^∗∗^*P* < 0.01 vs. control.

**FIGURE 2:**
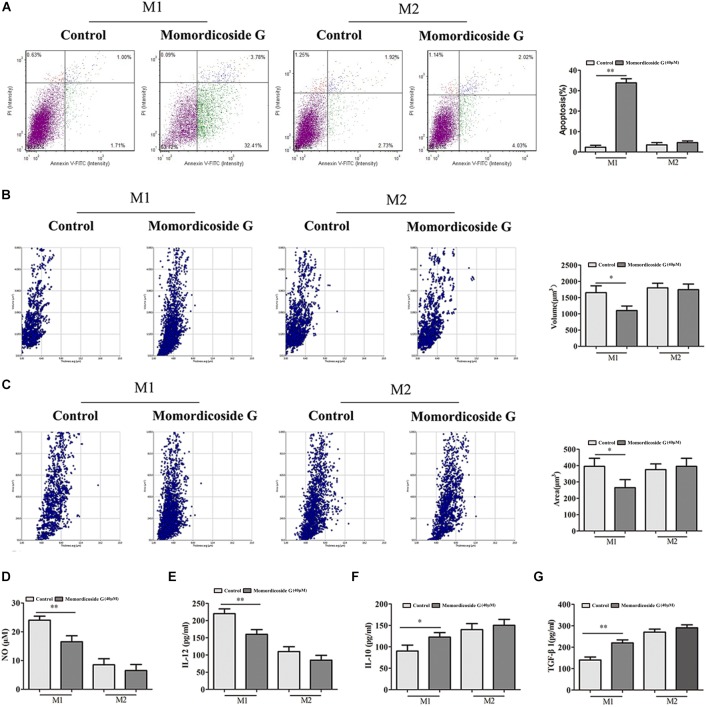
Momordicoside G induces M1 macrophage apoptosis *in vitro* **(A)** Cell apoptosis detected with Annexin V-FITC Apoptosis kit (*n* = 5). **(B,C)** Morphological changes analyzed with Laser holographic cell imaging and analysis system (*n* = 5, 20×). **(D)** NO (*n* = 5) **(E)** IL-12 (*n* = 5) **(F)** IL-10 (*n* = 5) **(G)** TGF-β1 (*n* = 5) The data presents mean ± SD, the experiments were repeated 3 times, and statistical significance was determined by a *t*-test. ^∗^*P* < 0.05, ^∗∗^*P* < 0.01 vs. control.

To explore the effects of momordicoside G on macrophages, we stimulated Raw264.7 macrophages with IL-10 to obtain the M2-like cells (arginase^+^ cells) and LPS to obtain the M1-like cells (iNOS^+^ cells) (Figure 1A). Momordicoside G at the dose of 10–40 μM had no effect on cell viability in M2-like macrophages but suppressed cell proliferation in M1-like macrophages in a dose independent manner (Figure 1B). Further, momordicoside G at the dose of 40 μM decreased ROS (Figure 1C) and promoted autophagy (Figure 1D,E) and thus induced apoptosis in M1-like macrophages (Figure 2A) with the changes of cell size and volume (Figure 2B,C). Cytokine assays further proved the effect of momordicoside G on M1-like macrophages, presenting as a decrease in the levels of NO and IL-12 and an increase in the levels of IL-10 and TGF-β1 (Figure 2D–G).

### Macrophage Infiltration Play an Important Role in Urethane-Induced Lung Tissue Injury and Carcinoma Lesions

**FIGURE 3:**
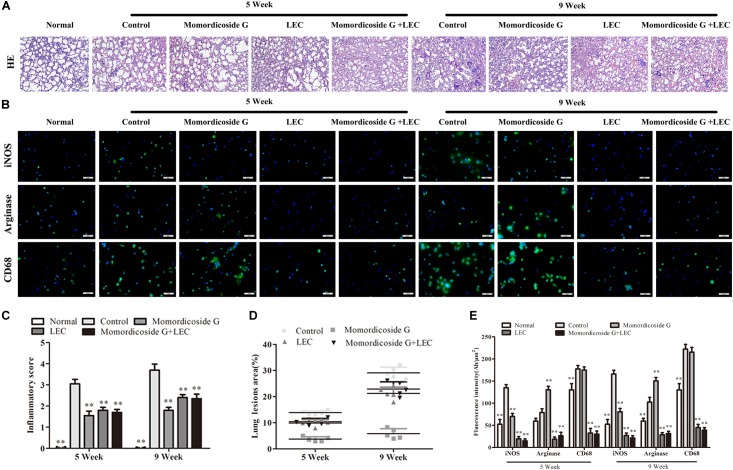
Momordicoside G prevents urethane-induced lung injury and carcinoma lesions. **(A)** Lung injury and carcinoma lesions examined by H&E staining (*n* = 10, 10×). **(B)** Lung alveolar cavity macrophage phenotypes indicated by immunofluorescence (*n* = 5, 40×). **(C)** Inflammatory score (*n* = 5). **(D)** The area of carcinoma lesions (*n* = 5). **(E)** The fluorescence intensity of different macrophage phenotypes (*n* = 5). The data presents mean ± SD, the experiments were repeated 3 times, and statistical significance was determined by a *t*-test. ^∗^*P* < 0.05, ^∗∗^*P* < 0.01 vs. control.

**FIGURE 4:**
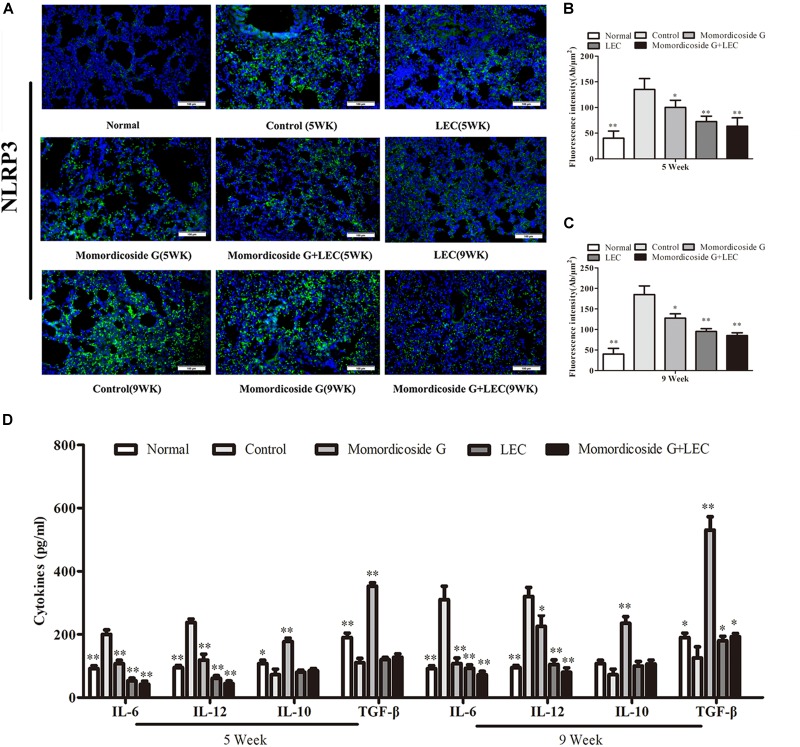
Momordicoside G affects inflammasome and cytokines during urethane-induced lung injury and carcinoma lesions. **(A)** NLRP3 inflammasome examined by immunohistochemistry (*n* = 5, 20×). **(B,C)** Fluorescence intensity (*n* = 5). **(D)** Cytokines in alveolar cavity (*n* = 5). The data presents mean ± SD, the experiments were repeated 3 times, and statistical significance was determined by a *t*-test. ^∗^*P* < 0.05, ^∗∗^*P* < 0.01 vs. control.

Urethane-induced mouse lung cancer is used for studying basic lung tumor biology and finding new tumor intervention strategies (Du et al., 2013). We found that carcinogenic time can controlled by the dose and frequency of urethane injection. In this study, urethane at the dose of 600 mg/kg body weight was injected intraperitoneally (i.p.) once a week. At five weeks, prior to lung carcinoma lesions, the four urethane injections led to obvious lung injury in control group (Figure 3A), presenting as an increase in lung inflammation (Figure 3C) and injury area (Figure 3D), which were positively correlated with macrophage infiltration (CD68^+^ cells) (Figure 3B). Immunohistochemical analysis showed that urethane induced obvious inflammasome, as indicated by staining for nucleotide binding oligomerization domain-like receptor protein 3 (NLRP3) in lung tissues (Figure 4A–C). At nine weeks, the eight urethane injections resulted in visible lung carcinoma lesions (Figure 3A) under a microscope accompanied by the aggravated macrophage infiltration (Figure 3B) and inflammasome (Figure 4A–C), but no lung cancer nodes were visible to the naked eye. These macrophages had high levels of iNOS^+^/CD68^+^expression (M1 like) (Figure 3B) and were consistent with the formed inflammasome (Figure 4A–C). ELISA showed that the levels of IL-10 and TGF-β1 (M2 cytokines) decreased and the levels of IL-6 and IL-12 (M1 cytokines) increased in the alveolar cavity in control mice compared to normal mice (Figure 4D). As expected, macrophage depletion by LEC following urethane injection significantly decreased lung tissue injury and carcinoma lesions (Figure 3A,C) accompanied by a reduction in NLRP3 inflammasome (Figure 4A–C).

### Momordicoside G Regulates Macrophages to Prevent Urethane-Induced Lung Injury and Carcinoma Lesions

To prove the preventive effects of momordicoside G on lung injury and carcinoma lesion, mice received momordicoside G after the first urethane injection. At five weeks, compared to the control group, momordicoside G treatment resulted in a reduction in lung injury (Figure 3A,C) accompanied by an increase in M2-like macrophages (arginese^+^ cells) (Figure 3B) and a decrease in inflammasome (Figure 4A–C) although it had little effect on total macrophages (CD68^+^ cells) (Figure 3B). At nine weeks, momordicoside G treatment resulted in not only mild lung tissue injury but also slight carcinoma lesions (Figure 3A,C) which were in line with the decreased M1-like macrophages (iNOS^+^ cells) (Figure 3B) and NLRP3 inflammasome (Figure 4A–C) and the increased levels of alveolar IL-10 and TGF-β1 (Figure 4D). The effects of momordicoside G on lung injury and carcinoma lesion were significantly attenuated by macrophage depletion (Figure 3, 4).

### Momordicoside G Exhibits Macrophage-Regulating Capacity in LPS-Induced Lung Injury Model

**FIGURE 5:**
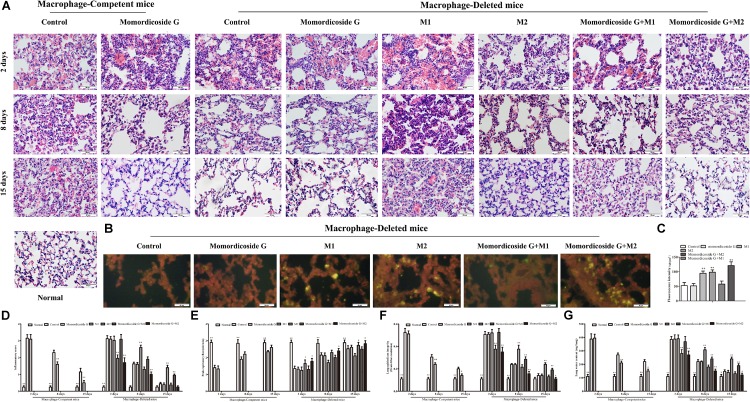
Momordicoside G promotes lung injury repair in LPS-induced lung injury model. **(A)** Lung injury by H&E staining in macrophage-competent and macrophage-deleted mice (*n* = 10, 40×). **(B)** CFSE–labeled M1 and M2 macrophages in lung sections from macrophage-deleted mice (*n* = 5, 40×). **(C)** Fluorescence intensity. **(D)** Inflammatory score (*n* = 5). **(E)** Peak expiratory flow (*n* = 5). **(F)** Lung epithelial integrity (*n* = 5). **(G)** Lung water content (*n* = 5). The data presents mean ± SD, the experiments were repeated 3 times, and statistical significance was determined by a *t*-test. ^∗^*P* < 0.05, ^∗∗^*P* < 0.01 vs. control.

**FIGURE 6:**
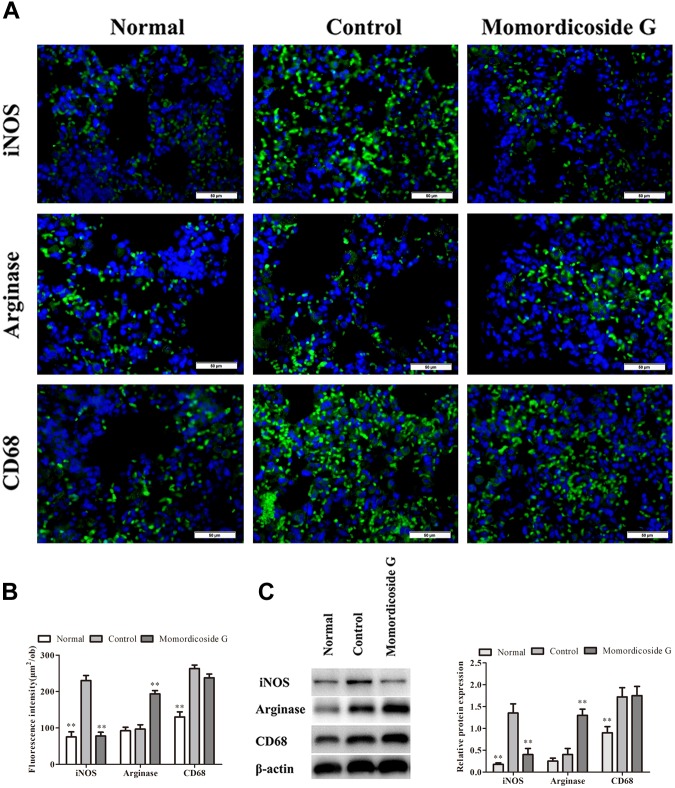
Momordicoside G suppresses M1 to maintain M2 macrophages in LPS-induced lung injury model. **(A)** Lung interstitial macrophage phenotypes indicated by immunofluorescence in lung sections from macrophage-competent mice (*n* = 5, 40×). **(B)** Fluorescence intensity score of lung interstitial macrophages (*n* = 5). **(C)** The protein expression of iNOS and arginase examined by Western blot in alveolar macrophages from macrophage-competent mice (*n* = 3). The data presents mean ± SD, the experiments were repeated 3 times, and statistical significance was determined by a *t*-test. ^∗∗^*P* < 0.01 vs. control.

To further prove the relativity of momordicoside G-ameliorated lung injury to M2 macrophages, we established an LPS-induced lung injury model in mice with macrophage-depletion or not and used momordicoside G alone or in combination with CFSE –labeled M1-like or M2-like Raw264.7 macrophages to treat the model mice. In macrophage-competent mice, momordicoside G could obviously improve lung injury (Figure 5A,D). In macrophage-deleted mice, the transfer of IL-10-stimulated M2-like macrophages ameliorated lung injury, whereas the transfer of LPS-stimulated M1-like macrophages exacerbated lung injury (Figure 5A,D). Momordicoside G alone did not significantly improve lung injury which was aggravated by M1-like macrophages and attenuated by M2-like macrophages (Figure 5A,D). The relativity of ameliorated lung injury to M2 macrophages was further confirmed in lung sections (Figure 5B). However, Momordicoside G decreased the injury-promoting efficacy of M1-like macrophages but synergistically improved lung injury when used in combination with M2-like macrophages accompanied by the improved lung function (Figure 5E) and lung permeability (Figure 5F,G). The M2-like macrophage dependent effect of momordicoside G on lung injury repair was further confirmed in lung sections (Figure 6A,B) in isolated alveolar macrophages by western blot in macrophage-competent mice (Figure 6C).

### Momordicoside G Regulates Macrophages by Targeting Multi-Protein Network

**FIGURE 7:**
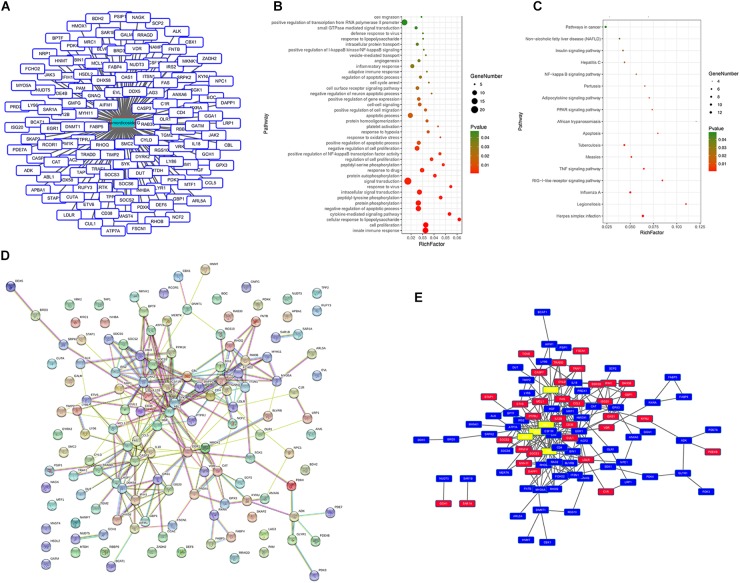
Momordicoside G regulates M1 macrophage by targeting multi-protein network. **(A)** The potential targets of Momordicoside G with docking score > 6.0. **(B)** GO enrichment analysis performed by DAVID and visualized by Omicshare. **(C)** KEGG enrichment analysis performed by DAVID and visualized by Omicshare. **(D)** The PPI network constructed by the STRING database. **(E)** The hub genes identified by Cytoscape.

We queried 590 up-regulated genes (LogFC ≥ 1.5, *P* < 0.05) and 994 down-regulated genes (LogFC ≤ −1.5, *P* < 0.05) related to M1-associated macrophages and obtained 181 targeting information. 136 of the potential targets with docking score > 6.0 (pKd/pKi) were selected for GO and KEGG analyses (Supplementary Table S1 and Figure 7A). The hypergeometric distribution count > 4 and *P* < 0.05 were set as threshold criteria to identify the functional gene ontology and pathway. GO enrichment analysis indicated that the potential targets of momordicoside G were primarily associated with the “signal transduction,” “innate immune response,” “cell proliferation,” “protein phosphorylation,” and “apoptotic process” terms (Figure 7B). KEGG enrichment analysis revealed that the potential targets of momordicoside G were significantly enriched in the “Pathways in cancer,” “TNF signaling pathway,” and “RIG-I-like receptor signaling pathway” terms (Figure 7C). The PPI network (Figure 7D) identified 4 key genes (JAK-2, ABL1, CASP3, and CBL), which were hub genes for momordicoside G (Figure 7E).

## Discussion

Macrophages are primary phagocytic cells and are also major regulatory cells for tissue homeostasis and injury repair (Lavin et al., 2015). Macrophages can be generally divided into classically activated M1-like macrophages and alternatively activated M2-like macrophages (Mcwhorter et al., 2013). In physiology, lung injury first stimulated M1-like macrophage polarization and later alternatively polarized M2-like macrophages (Sun et al., 2016). It has been reported that the adoptive transfer of M2-like macrophages could accelerate lung repair and reduce lung inflammation as well as lung injury in mice (Tu et al., 2017). In contrast, the depletion of M2-like macrophages exaggerates acute lung injury in mice (Kambara et al., 2015). Consistent with these reports, in this study, urethane-induced lung injury was positively correlated with the number of M1-like macrophages and was negatively correlated with the number of M2-like macrophages, whereas macrophage depletion could significantly decrease lung tissue injury and carcinoma lesions, indicating an important role of macrophage phenotypes in lung tissue injury and carcinoma lesions. In addition, in this study, momordicoside G treatment resulted in less lung tissue injury and carcinoma lesions accompanied by the decreased M1-like macrophages and the increased M2-like macrophages, whereas macrophage depletion attenuated the protective effect of momordicoside G on lung injury, suggesting a crucial contribution of the retained M2 macrophages to momordicoside G-affected lung injury and carcinoma lesions.

Recently, *M. charantia* consumption increased due to pre-clinical anti-cancer efficacy studies demonstrating bitter melon potential to inducing apoptosis, cell cycle arrest, autophagy and inhibiting cancer stem cells (Dandawate et al., 2016; Gu et al., 2017). Although this plant has high ethnopharmacological value for treating various diseases, anti-inflammatory efficacy should be common mechanisms (Dandawate et al., 2016; Shuo et al., 2017). The injury and cancerization are linked (Tolg et al., 2014). The injury creates a susceptible microenvironment for cancer initiation, and cancerization reflects the aberrant physiological response to tissue injury (Steiling et al., 2008; Quail and Joyce, 2013). In this study, urethane-induced lung injury was positively correlated with M1-like macrophage infiltration. Considering a cancer-promoting effect of inflammation (Morrison, 2012), we think that M1-like macrophages exert an inflammation-promoting function to prevent injury repair and therefore stimulate carcinoma lesions. Based on the fact that M2-like macrophages exert a protective action on injury, we believe that regulating macrophage phenotypes rather than complete depletion of macrophages is a better approach for injury repair and cancer prevention. In LPS-induced lung injury model, momordicoside G did not improve lung injury in macrophage deleted mice but had synergistical efficacy when used in combination with M2-like macrophages, suggesting a need for M2 macrophage retention. In addition, *in vitro*, momordicoside G decreased ROS and promoted autophagy and thus induced apoptosis in M1-like macrophages but not in M2-like macrophages, indicating a reciprocal inhibition between M1 and M2 cells as well as a selective effect of momordicoside G on macrophage phenotypes. This finding may explain *M. charantia* consumption against diseases associated with aberrant and uncontrolled inflammation.

Although *M. charantia* and its extracts have been extensively investigated, there is insufficient information related to the mechanism by which *M. charantia* and its extracts exert their anticancer properties in different cancers (Nerurkar and Ray, 2010). It has been proved that *M. charantia* and its extracts can modulate AKT/mTOR/p70S6K signaling, cell cycle regulatory proteins and apoptosis-associated proteins in different cancers (Farooqi et al., 2018). Minari et al. (2018) revealed that there may be possibility of prevention of damage to k-ras gene as a result of the effect of *M. charantia* extract. In this study, we try hard to explain the mechanism by which momordicoside G regulates macrophage phenotypes using bioinformatics analysis and found that the potential targets of momordicoside G regulating macrophages were primarily associated with the “signal transduction,” “innate immune response,” “cell proliferation,” “protein phosphorylation,” and “apoptotic process,” indicating a function of momordicoside G targeting multi-protein network, such as “TNF signaling pathway” and “RIG-I-like receptor signaling pathway,” whereby it regulates macrophage phenotypes to prevent the injury and cancerization. Further mechanism studies are necessary to verify its medicinal applications.

As is the case for other types of cancer, the normal lung cells spend several years transforming into early precursor lesions and finally into lung cancer, which provides us with several opportunities for preventing the progression of precancerous lesions (Gazdar et al., 2011). Although some studies showed a therapeutic effect of *M. charantia* and its compounds on the formatted tumors (Nerurkar and Ray, 2010), we believe that momordicoside G may produce better curative effects as a chemopreventive agent than as a chemotherapeutic agent. Although this study focused on carcinogen-induced mouse lung precancer, based on macrophage regulation, it is likely that momordicoside G and *M. charantia* mediate supplementary benefits for cancer prevention, including reduced chronic inflammation and enhanced injury repair. In addition, momordicoside G as an edible ingredient, logically, its preventive efficacy on lung injury and carcinoma lesions may be overstated in rodent or cell models. With cancer being a global issue and understanding the need for activation of patient’s innate antineoplastic agents, momordicoside G may provide efficient opportunities as a chemoprevention agent targeting multi-protein network. Certainly, there are visible knowledge gaps related to *M. charantia* and active components in cancer prevention, we need yet develop comprehensive understanding of *M. charantia* and its active components mediated regulation of signal transduction pathways.

In summary, M2-like macrophages play an important role in wound healing and tissue homeostasis, our findings suggest several potential clinical implications. First, there will not be carcinogenesis if tissue injury can be timely and completely cured. Second, Momordicoside G can selectively suppress M1 to maintain M2 macrophages for lung injury repair and therefore prevent inflammation-related lung carcinogenesis. Third, the combination of momordicoside G and M2 macrophages synergistically promote lung injury repair and can be a novel strategy for cancer chemoprevention.

## Author Contributions

GD conceived and designed the experiments. GD, ZD, SZ, YL, LZ, YW, GY, MZ, and MW performed the experiments. GD, ZD, and SZ analyzed the data. GD, JL, QT, and YD contributed to reagents, materials and analysis tools. GD, ZD, and SZ wrote the manuscript and plotted the results.

## Conflict of Interest Statement

The authors declare that the research was conducted in the absence of any commercial or financial relationships that could be construed as a potential conflict of interest.

## Abbreviations

AO: acridine orange
ANOVA: analysis of variance
BSA: bovine serum albumin
DAPI: diamidino-phenylindole
HRP: horseradish peroxidase
hs-CRP: hypersensitive C-reactive protein
IL-6: interleukin-6
IL-10: interleukin-10
IL-12: interleukin-12
LEC: liposome-encapsulated clodronate
LPS: lipopolysaccharide
MTT: thiazole blue
NLRP3: oligomerization domain-like receptor protein
NO: nitric oxide
ROS: reactive oxygen species
SDS: sodium dodecyl sulfate
TGF-β1: transforming growth factor-β1.
