# Supplementary material for: Momordicoside G Regulates Macrophage Phenotypes to Stimulate Efficient Repair of Lung Injury and Prevent Urethane-Induced Lung Carcinoma Lesions
Source: Front Pharmacol. 2019 Mar 29;10:321. doi: 10.3389/fphar.2019.00321 (PMC6450463; doi:10.3389/fphar.2019.00321)
Supplement: Supplementary file 1 [file Table_1.DOCX]

| **Up-regulated genes** | | | | | | | | |
| --- | --- | --- | --- | --- | --- | --- | --- | --- |
| Protein Name | PDB ID | Docking Scores (pKd/pKi) | Protein Name | PDB ID | Docking Scores (pKd/pKi) | Protein Name | PDB ID | Docking Scores (pKd/pKi) |
| IL12B | 3D85 | 6.076 | IFIH1 | 3GA3 | 7.506 | GCH1 | 1A8R | 8.244 |
| VRK2 | 2V62 | 6.094 | RUFY3 | 2DWK | 7.54 | MYH11 | 1BR2 | 8.25 |
| LDLR | 2FCW | 6.164 | STAP1 | 3MAZ | 7.563 | MTF1 | 1I4W | 8.251 |
| DAPP1 | 1FAO | 6.265 | OAS1 | 1PX5 | 7.674 | PTPRJ | 2NZ6 | 8.263 |
| ICAM1 | 1IAM | 6.386 | DHX58 | 3EQT | 7.771 | TRAF1 | 3M0D | 8.269 |
| TRADD | 1F3V | 6.542 | AIM1 | 3CW3 | 8.007 | CUL1 | 1LDJ | 8.28 |
| CCL5 | 1B3A | 6.569 | RAB30 | 2EW1 | 8.007 | JAK2 | 3KRR | 8.285 |
| FSCN1 | 3LLP | 6.599 | ISG20 | 1WLJ | 8.04 | TGM2 | 2Q3Z | 8.298 |
| MAST4 | 2W7R | 6.637 | BOC | 3N1M | 8.042 | PDE4B | 2CHM | 8.302 |
| ETV6 | 1JI7 | 6.677 | UBE2S | 1ZDN | 8.064 | RBBP9 | 2QS9 | 8.325 |
| INHBA | 2ARP | 6.682 | VDR | 1IE9 | 8.103 | CYLD | 2VHF | 8.328 |
| DDX58 | 3LRR | 6.747 | PPM1K | 2IQ1 | 8.105 | CD38 | 3F6Y | 8.335 |
| LAG3 | 2FO1 | 6.802 | C1R | 2QY0 | 8.178 | KYNU | 3E9K | 8.347 |
| SOCS3 | 2HMH | 6.919 | PAM | 2DOH | 8.194 | TAP1 | 2IXE | 8.365 |
| SOCS2 | 2C9W | 6.956 | NAMPT | 3DHF | 8.201 | JAK3 | 3LXL | 8.375 |
| SAR1A | 2GAO | 7.113 | CASP3 | 2DKO | 8.213 | MCL1 | 2NL9 | 8.376 |
| FAS | 3HHD | 7.314 | CASP7 | 2QL9 | 8.223 | GBP1 | 1F5N | 8.417 |
|  |  |  |  |  |  |  |  |  |
| **Down-regulated genes** | | | | | | | | |
| Protein Name | PDB ID | Docking Scores (pKd/pKi) | Protein Name | PDB ID | Docking Scores (pKd/pKi) | Protein Name | PDB ID | Docking Scores (pKd/pKi) |
| PSIP1 | 2B4J | 6.252 | SCP2 | 2SAS | 7.104 | FABP5 | 1B56 | 8.19 |
| ALK | 3L9P | 6.293 | RHOB | 2FV8 | 7.135 | ABL1 | 3KFA | 8.192 |
| AP2S1 | 2VGL | 6.306 | DUT | 1EUW | 7.136 | MTDH | 1H5Q | 8.201 |
| GALM | 3MWX | 6.349 | DNMT1 | 3EPZ | 7.149 | GLYR1 | 2UYY | 8.207 |
| MRC1 | 1DQG | 6.381 | APBA1 | 1AQC | 7.252 | NPC1 | 3GKJ | 8.232 |
| FCHO2 | 2V0O | 6.382 | DEF6 | 1ZMQ | 7.264 | HSDL2 | 3KVO | 8.247 |
| ANXA6 | 1M9I | 6.383 | RHOQ | 2ATX | 7.287 | PDK3 | 1Y8O | 8.257 |
| HGF | 3HMS | 6.419 | PDXK | 2YXT | 7.336 | HNMT | 2AOT | 8.261 |
| NRP1 | 2QQI | 6.485 | ATP7A | 3CJK | 7.401 | SOCS6 | 2VIF | 8.261 |
| ARL5A | 2H17 | 6.52 | NAGK | 2AP1 | 7.648 | ADK | 2ABS | 8.265 |
| SKAP2 | 1U5F | 6.523 | LY96 | 2E56 | 7.665 | LY86 | 3M7O | 8.27 |
| DDX5 | 3FE2 | 6.567 | FNTB | 2H6F | 7.88 | DYRK2 | 3KVW | 8.273 |
| ITSN1 | 3FIA | 6.573 | LRP1 | 1J8E | 7.885 | CAT | 1Q23 | 8.275 |
| GMFG | 1VKK | 6.59 | GPX3 | 2R37 | 7.893 | HMOX1 | 1N45 | 8.276 |
| MKNK1 | 2HW6 | 6.603 | NCF2 | 1HH8 | 7.929 | GNAQ | 3AH8 | 8.279 |
| CBX1 | 2FMM | 6.604 | RRAGD | 2Q3F | 7.983 | MERTK | 3BRB | 8.291 |
| IRS2 | 3BU3 | 6.619 | RGS10 | 2IHB | 8.059 | BLVRB | 1HDO | 8.292 |
| BPTF | 2RI7 | 6.654 | GATM | 1JDW | 8.067 | EVL | 1QC6 | 8.292 |
| RAC2 | 2W2T | 6.66 | SYK | 3E9H | 8.076 | AIFM1 | 1M6I | 8.311 |
| PRDX1 | 3HY2 | 6.691 | BCAT1 | 2COI | 8.097 | CSF1R | 2I1M | 8.347 |
| BRD3 | 2NXB | 6.73 | CUTA | 1KR4 | 8.106 | MYO5A | 1W7J | 8.357 |
| CD4 | 2NY1 | 6.735 | RCOR1 | 2IW5 | 8.111 | SMC2 | 3L51 | 8.363 |
| GGA1 | 1J2J | 6.89 | CBL | 3BUX | 8.126 | SRPK2 | 2X7G | 8.364 |
| EGR1 | 1LLM | 6.953 | ZADH2 | 2C0C | 8.132 | SGK1 | 2R5T | 8.366 |
| SAR1B | 1F6B | 6.987 | OLR1 | 1YPQ | 8.149 | BDH2 | 2AG5 | 8.368 |
| RARA | 1DSZ | 6.995 | NUDT5 | 2DSC | 8.151 | PDK4 | 2E0A | 8.382 |
| RXRA | 1DSZ | 6.996 | PDE7A | 1ZKL | 8.161 | TPP2 | 3LXU | 8.393 |
| NUDT3 | 2FVV | 7.065 | TIMP2 | 2E2D | 8.166 |  |  |  |
| IL18 | 2VXT | 7.087 | FABP4 | 2HNX | 8.167 |  |  |  |
